# The pathway to child survival in the Birhan Cohort, Ethiopia, 2018–22

**DOI:** 10.7189/jogh.15.04270

**Published:** 2025-10-03

**Authors:** Negalign Berhanu Bayou, Tsinuel Girma Nigatu, Biruk Hailu Tesfaye, Bezawit Mesfin Hunegnaw, Clara Pons-Duran, Kassahun Alemu, Lisanu Taddesse, Delayehu Bekele, Getachew Tolera, Grace Chan

**Affiliations:** 1 Health System and Reproductive Health Research Directorate, Ethiopian Public Health Institute, Addis Ababa, Ethiopia; 2 Department of Health Policy and Management, Institute of Health, Jimma University, Jimma, Ethiopia; 3 The University of British Columbia, Addis Ababa, Ethiopia; 4 Maternal, Child and Adolescent Health lead Executive Office, Ministry of Health, Ethiopia; 5 Department of Pediatrics and Child Health, St. Paul’s Hospital Millennium Medical College, Addis Ababa, Ethiopia; 6 HaSET Maternal and Child Health Research Program, Addis Ababa, Ethiopia; 7 Department of Epidemiology, Harvard University T.H. Chan School of Public Health, Boston, Massachusetts, USA; 8 Department of Gynecology and Obstetrics, St. Paul’s Hospital Millennium Medical College, Addis Ababa, Ethiopia; 9 Research and Technology Transfer Directorate, Ethiopian Public Health Institute, Addis Ababa, Ethiopia; 10 Department of Pediatrics, Children’s Hospital of Philadelphia, Perelman School of Medicine, University of Pennsylvania, Philadelphia, Pennsylvania, USA

## Abstract

**Background:**

Child mortality remains a concern in Ethiopia despite the significant achievements in the past three decades. Proper implementation of the existing low-cost interventions can prevent two-thirds of the deaths. Understanding illness recognition, care-seeking behaviours, and barriers that caregivers encounter during a child’s illness along care pathways is imperative. We aimed to describe illness recognition and reactions of caregivers of children <2 years, and factors associated with severe illness or death related to the care pathways, including the child, caretaker, household, and health system.

**Methods:**

We conducted a prospective cohort study using an open birth cohort of Birhan field site, from December 2018 to November 2022. The analysis included newborns followed up to two years old who had an illness episode and for whom data were available for the mother-child *dyad*. We extracted and linked data on community follow-up and morbidity visits, clinical signs and symptoms of illness at health facility visits, verbal autopsy of deceased children, and maternal health and healthcare. We used descriptive and logistic regression analyses.

**Results:**

Of 3969 eligible children enrolled in the Birhan Cohort, 1397 (37.8%) had at least one episode of illness during the first two years of life. Of those, 108 (8%) experienced a severe illness or died, of which the majority (n = 76; 70.4%) were newborns. Most sick children (n/N = 714/1187) did not get treatment from a formal source; 53.1% (n/N = 684/1289) of those with mild or moderate illness and 27.8% (n/N = 30/108) of the severely ill or deceased. The mean delay in care-seeking was 5.9 (standard deviation (SD) = 10.6) days for those with mild or moderate illness, and 1.7 (SD = 0.58) for the severely ill or deceased. Only 4.8% (n/N = 27/559) of children sought care from a health post (HP), and 68.1% (n/N = 94/138) of children were referred for further care. Only 68.4% (n/N = 13/19) of the severely ill or deceased children were referred, of which 3 (4.9%) accepted the referral. Compared to a newborn, being a young infant (adjusted odds ratio (aOR) = 0.05; 95% confidence interval (CI) = 0.008–0.27) and a child (aOR = 0.03; 95% CI = 0.005–0.17) were associated with a reduction in the odds of severe illness or death. Children who sought care from an HP had a higher risk of severe illness or death than those who consulted a government hospital (aOR = 19.6; 95% CI = 2.71–142.40). Belonging to a rich family resulted in a reduction in the odds of the outcome compared to a poor household (aOR = 0.15; 95% CI = 0.02–0.94).

**Conclusions:**

Illness recognition and care-seeking were low in the Birhan field site, and when care was sought, it was delayed. Care was sought from an HP in rare cases. Health workers did not refer about a third of severely ill or deceased children for further care. Being a newborn, consulting a HP rather than a hospital, and belonging to a poor family had a significantly higher risk of severe illness or death. Strategies should be devised targeting the modifiable factors identified at individual, family, or community and health facility levels to improve child survival.

The under-five mortality rate has declined in Ethiopia by two-thirds, from 204 in 1990 to 55 in 2019 per 1000 live births [1]. Although causes vary by age group, pneumonia, diarrhoea, and newborn conditions of asphyxia, sepsis, and prematurity account for nearly 90% of under-five deaths in Ethiopia. The underlying cause for almost half of the under-five deaths in the country is under-nutrition [2]. However, demographic, socioeconomic, and behavioural variables are known to influence child survival in the country [3].

Proper implementation of existing low-cost interventions can prevent two-thirds of under-five deaths [4]. Only 21% of sick children in sub-Saharan African countries receive effective healthcare services [5]. The Federal Ministry of Health of Ethiopia/Maternal and Child Health Directorate prioritised a package of high-impact and cost-effective newborn and child survival interventions for 2015–16 to 2019–20 [2]. These include integrated community management of childhood illnesses (*i.e.* pneumonia, diarrhoea, malaria, and malnutrition), community-based newborn care, and community-based nutrition. These services are delivered in a continuum of care approach, or care pathway, at household/community and individualised clinical care levels. This care pathway enhances quality of care across the continuum by improving risk-adjusted patient outcomes, patient satisfaction, and efficient resource use [6]. Health posts (HPs), health centres (HCs), and primary hospitals are service delivery points in rural settings, while HCs provide basic and emergency care for communities as the first entry point to the health system, and hospitals serve as referral facilities in urban settings. The Health Development Army platform, a network of five households and one model family to influence one another in practising healthy behaviour, is used to empower and engage communities [2].

In the care pathway, understanding illness recognition, care-seeking behaviours, and barriers that caregivers encounter during a child’s illness has become increasingly recognised [7]. The success of a care pathway can be affected by individual, family, and community-level factors [8]. Therefore, besides the knowledge of biological and clinical causes of child death, information about challenges at household, community, and health system levels is critical to improve interventions to avert morbidity and mortality [8].

However, rigorous analytic evidence on care pathways is scarce in Ethiopia. Previous studies on sick childcare practice in the country have generated fragmented evidence, mainly focussing on the prevalence and determinants of child mortality, and the prevalence and patterns of health-seeking behaviour of caretakers. Only two studies [9,10] analysed care pathways. Due to the cross-sectional design, descriptive analysis approach, and focussing narrowly on clinical pathway analysis at the HP care level, the evidence they generated is not rigorous and comprehensive. The care pathways’ bottlenecks have not been identified or linked to quantified risks of severe illness or mortality. Therefore, we aimed to describe how caregivers of children <2 years recognise and respond to illness, as well as the factors related to the child, caregiver, household, and health system associated with severe illness or death along the care pathway.

## METHODS

### Study design

We analysed secondary data from the Birhan Cohort collected from May 2018 to November 2022. Data collection started in December 2018 with pregnancy enrollments and ended in March 2020. The last child enrolled in the cohort was born in November 2020. Therefore, the follow-up ended in November 2022 when all enrolled children reached their second birthday. Newborns were followed at birth (zero days), sixth, 28th, and 42nd day, while older children were followed at sixth, 12th, and 24th month. Data on labour and delivery and immediate newborn care were collected for those enrolled within 24 hours of birth. Caretakers were interviewed at sick visits to a health facility on clinical signs and symptoms of illness. Sick newborns and older children in the community were visited, and caretakers were interviewed. More details on the study design are described in a published paper [11]. A nested verbal autopsy (VA) study collected data after six weeks of a child’s death [11] using the standard 2016 World Health Organization VA questionnaire [12]. Cause-of-death related information was generated through the InterVA process.

### Study site

The Birhan field site was established in 2018 to determine the magnitude and causes of morbidity and mortality among women and children in Ethiopia. Located in the North Shewa Zone of the Amhara Region, 130 km north of Addis Ababa, it serves as a research and training platform. The site covers two districts, Angolela Tera and Kewet/Shewa Robit. According to the initial 2018 census, a total of 77 766 people resided in 18 933 households [13].

### Study population

All children <2 years enrolled in the Birhan Cohort, along with their primary caregivers, served as a study population. We included all children who had at least one follow-up contact after birth, who had an illness episode, or who died and for whom data were available for the mother-child *dyad*. To screen eligible children, we used health status since the last visit (every three months), which applied to scheduled quarterly visits, and sickness in the previous two weeks, which applied to new enrolments and unscheduled morbidity visits.

### Variables and measurements

#### Outcome

We used a composite of risks of severe illness or death among children under two associated with sickness-to-care pathways as the outcome. Since the number of observations for each round of visits did not allow for inferential analysis separately for the different time points, we created the composite outcome for each child using data for all visits they made during the follow-up period. We combined severe illness and mortality for the same reason. Evidence shows that the mechanisms that lead to death and life-threatening conditions among children are similar [14]. It is assumed that deceased children were seriously ill, allowing analysis of care-seeking and provision experiences, although it is likely that a few children might experience death [7].

Trained professional midwives captured VA data through the routine surveillance system. The likely cause of death was ascertained using the InterVA algorithm, while two local physicians ascertained the likely diagnosis or cause of illness and illness severity.

We derived data on illness severity from the symptoms reported by caretakers during neonatal and child community morbidity visits and from the VA. We used an illness severity scoring system adapted from Koffi et al. [15], which is based on the reported feeding behaviours and mental status of the children. These parameters reflect children’s genuine illness severity and caretakers’ perception of illness severity and healthcare-seeking [16]. The scoring system has been used in previous similar studies for various age groups [15,17]. Physicians classified a child’s condition as either severe or non-severe illness.

#### Exposures

The exposure variables included child, caretaker/household/family, and health system-related factors. Child-related exposures included sex, age, type of illness/diagnosis, birth weight (low birth weight (LBW) *vs.* non-LBW), and gestational age at delivery (preterm (<37 weeks) *vs.* term). Caretaker/household/family-related exposures included relationship of child to household head, rural residence, lack of formal education, poverty, care-seeking for sick child, non-use of maternal healthcare during pregnancy, and labour and delivery of the index child (*e.g.* antenatal care, facility birth, care for maternal complications, non-compliance to referral advice, maternal age, marital status; priory pregnancy history of early neonatal death, premature birth, LBW, early neonatal jaundice, birth defects; and past medical history of malaria, anemia, diabetes, (pre)eclampsia, sexually transmitted infections, cardiac and renal diseases, tuberculosis, and cancer). Health system-related exposures included distance to the nearest health facility >30-minute, type of care provider (if formal care was sought), and a child with danger signs or symptoms not referred for better care. We measured time to consultation as the number of days between the onset of illness and the first consultation of formal care, if such care was sought.

### Data

We extracted the required data from all domains from the Birhan Cohort. We extracted background information about households and caretakers (if the mother was not a caretaker) from the census data sets. Similarly, we obtained data on both community or facility follow-up and morbidity of children <2 years from caretakers’ interviews captured through the Birhan Cohort. The key data from this source included sociodemographic characteristics of the child, signs and symptoms of illness, essential elements of the care-seeking process such as recognition of symptoms of illness, whether adequate care was provided, whether and what type of outside-the-home care was sought (informal or formal source), delays to formal healthcare-seeking, and compliance with referral and treatment advice. We also obtained past maternal history and healthcare-seeking of the mother, including complications during pregnancy, labour, and delivery of the index child, from the same source. We obtained similar data from the VA data set for the deceased children. We linked data from all the sources for each child using a unique child identifier, which was connected with the identifiers of caretakers, household, and health facilities.

### Analysis

We imported the extracted data into Stata, version 17.0 (StataCorp LLC, Texas, USA), then cleaned and linked by child for analysis. The analysis was based on the Pathway to Survival Conceptual Framework (PSCF), which was first developed in 1996 by Waldman and colleagues [8] to support the implementation of the integrated management of childhood illness approach for addressing the prevalence of different social and non-biological child mortalities and the related barriers. Relative to other frameworks, PSCF is more comprehensive in capturing evidence. For example, it addresses all determinants suggested in The Three Delays Model, which is primarily focussed on delays in healthcare services and their barriers for case management at each of the three stages [18]. The PSCF organises steps that families, communities, and health systems must take to prevent illness and treat sick children and provides a valuable template for investigating the interaction between community and health system. It outlines the steps necessary to restore a sick child to health and survival [8]. We applied the framework with the following assumptions.

In this study, illness was recognised at home or in the community by caretakers, health extension workers, community informants, or the Birhan research team through active or passive surveillance. It could also be recognised at a health facility by health workers. Once illness was recognised, care could be provided at home or sought from outside of the home, which could be from informal or formal sources. Informal care could be sought from traditional healers or religious sources. Formal healthcare could be sought either from public or private providers. Caretakers could provide home care before seeking any outside care, or they could opt to pursue formal care first and then switch to informal sources depending on the outcome or perceived quality of care received.

The PSCF also identifies that the role of enabling factors for successful implementation of priority interventions, such as training and quality of care provided, including laboratory capacity of health facilities, is also critical in the care pathway to child survival. It acknowledges that child, caretaker, and family/household-related factors can also determine a child’s progression along the care pathway. However, wellness and preventive care provided both inside the home (*e.g.* breastfeeding) and outside the home (*e.g.* immunisation) were beyond our scope.

We computed descriptive statistics using frequencies and proportions for the measured variables. We estimated the proportions of severe illnesses or deaths. We carried out logistic regression analysis for the composite outcome, as separate modelling for each follow-up time point was not possible due to the small number of observations. We used odds ratios (ORs) and 95% confidence intervals (CIs) to determine the strength of associations. In the final multivariate model, we included exposure variables that showed a statistically significant association (with the outcome at *P* < 0.05) in the univariate analysis, using a complete case analysis. We conducted a sensitivity analysis to identify the effects of the confounders and covariates on the validity of the conclusions. We excluded exposure variables with evidence of multicollinearity – defined as a tolerance value of ≤0.1 or a variance inflation factor of ≥10.

### Operational definitions

We classified a child as having a severe illness if they had a ‘yes’ response for one or both of the following danger signs: inability to feed or suck, or being very sleepy or unconscious with decreased movement. If a child experienced mild, moderate, or severe illness episodes at the same or different time points, we counted them only once under severe illness. Similarly, if a severely ill child had died, we considered them deceased. We considered a weight of <2500 g at birth as LBW.

We adopted the definitions of caretaker’s recognition of severe illness from the World Health Organization guidelines for integrated management of childhood illness [19]. Informal care refers to any care delivered by a provider who was not a public or private health facility or a trained community health worker. This included care received from traditional healers or religious intervention. Inside home treatment was defined by the caretakers’ mention of at least one of the actions taken depending on the type of illness encountered, such as oral rehydration solution, more fluid and/or food, drugs (*e.g.* antipyretic/antipain), and herbal medicine.

We constructed a wealth index score using principal components analysis. The index accounted for differences across urban and rural settings. We categorised the score into tertiles for the households relative to the wealth of the study area. We considered households in the first tertile as poor, those in the second as middle-income, and those in the third tertile as wealthy households.

## RESULTS

### Characteristics of the study participants

Of the 4354 enrolled caretakers, 2682 (61.1%) lived in Kewet *woreda* and 3544 (81.4%) lived in rural settings. Households with 4–6 children made up 50.9% (n/N = 1890/3715) of the study population. Of the 4354 enrolled families, 35.1% were classified as poor, 34.7% as middle income, and 30.2% as rich. To reach the nearest HC or hospital, 1005 (23.1%) of families travelled <30-minute and 1074 (24.7%) travelled ≥2 hours. Of the 4354 caretakers, 55.4% were aged 20–24 years, and 7.5% were >20 years old. Most (n/N = 4045/4313; 94%) were married, 90.9% (n/N = 3952/4346) were Amhara by ethnicity, 72.8% (n/N = 3064/4211) were housewives, and 49.8% (n/N = 2166/4346) received formal education (**Table 1**).

**Table 1:** Household characteristics of caretakers enrolled in the maternal and birth cohort of the Birhan Health and Demographic Surveillance System, North Shoa Zone, Amhara Region, Ethiopia, 2018–22

Characteristics	n (%)
Woreda (n = 4354)	
*Angolela Tera*	1672 (38.4)
*Kewet*	2682 (61.6)
Residence (n = 4354)	
*Urban*	810 (18.6)
*Rural*	3544 (81.4)
Family size (n = 3715)	
*1–3*	1294 (34.8)
*4–6*	1890 (50.9)
*7 or more*	531 (14.3)
Wealth group (n = 4354)	
*Poor*	1529 (35.1)
*Medium*	1509 (34.7)
*Rich*	1316 (30.2)
Estimated walking time to reach the nearest HC or hospital in minutes (n = 4354)	
*<30*	1005 (23.1)
*30–60*	1220 (28.0)
*60–120*	1055 (24.2)
*≥120*	1074 (24.7)
Caretakers’ age group (n = 4354) in years	
*Below 20*	327 (7.5)
*20–29*	2411 (55.4)
*30–39*	1378 (31.7)
*40–49*	164 (3.8)
*50 and above*	74 (1.7)
Ethnicity (n = 4346)	
*Amhara*	3952 (90.9)
*Oromo*	232 (5.3.)
*Argoba*	158 (3.6.)
*Other*	4 (0.1)
Religion (n = 4347)	
*Orthodox Christian*	3446 (79.3.)
*Muslim*	884 (20.3.)
*Protestant Christian*	17 (0.4.)
Marital status (n = 4313)	
*Single (never married)*	118 (2.7)
*Married*	4045 (93.8)
*Divorced/separated*	125 (2.9)
*Widowed*	25 (0.6)
Education (n = 4346)	
*Formal schooling*	2166 (49.8)
*Informal schooling (literacy campaign or religious school)*	88 (2.0)
*No schooling*	2092 (48.1)
Occupation (n = 4211)	
*Housewife*	3064 (72.8)
*Farmer*	997 (23.7)
*Student*	87 (2.1)
*Daily laborer*	54 (1.3)
*Other*	9 (0.2)

HC – health centre

Of the enrolled children, 51.6% (n/N = 2237/4336) were males, and 72.5% (n/N = 2713/3744) were aged 12–24 months. Most children were related to the household heads as a son or a daughter (n/N = 2502/2587; 96.7%), 95.1% (n/N = 4142/4354) weighed ≥2500 g at birth, and 26.5% (n/N = 685/2587) were born in the community (**Table 2**).

**Table 2:** Characteristics of children enrolled in the Birhan Cohort, 2018–22

Characteristics	n (%)
Sex (n = 4336)	
*Male*	2237 (51.6)
*Female*	2099 (48.4)
Age group (n = 3744)	
*Newborn*	100 (2.7)
*Infant (28 days–1 year)*	931 (24.9)
*Older child (12–24 months)*	2713 (72.5)
Relationship to household head (n = 2587)	
*Son or daughter*	2502 (96.7)
*Other (e.g. grandchild, sister/brother, nephew/niece, not related)*	85 (3.3)
Birth weight (n = 4354)	
*2500 g or above*	4142 (95.1)
*Below 2500 g*	212 (4.9)
Gestational age (n = 2295) in weeks	
37 or above	1793 (78.1)
Below 37	502 (21.9)
Birthplace (n = 2587)	
*Community*	685 (26.5)
*Facility*	1902 (73.5)

### Illness recognition

Of the 3969 children <2 years for whom data were available for the mother-child *dyad*, 1397 (37.8%) had at least one episode of illness. Caretakers reported that 15.8% (n/N = 195/1311) of the children had one or both general danger signs (*i.e.* unable to feed or suck and sleepy or lethargic). The remaining 1039 (84.2%) children had mild or moderate illness, with none of the danger signs reported.

**Figure 1:**
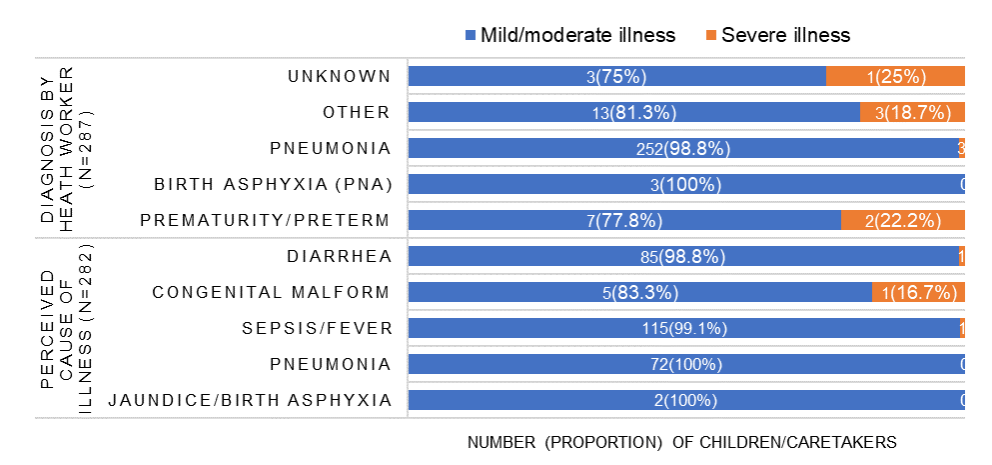
Illness severity of children enrolled in the birth cohort 2018–22.

As a cause of illness, 115 (41.1%) of caretakers frequently mentioned fever or sepsis, followed by diarrhoea (n = 85; 30.5%), pneumonia (n = 72; 25.5%), and jaundice/birth asphyxia (n = 2; 0.7%). Similarly, 252 (88.8%) health workers diagnosed pneumonia as the leading cause of illness. Three of the children (1.18%) with a pneumonia diagnosis progressed to severe illness or death. Three of nine children had a diagnosis of prematurity or preterm birth, of which two were severely ill or deceased (**Figure 1**).

**Figure 2:**
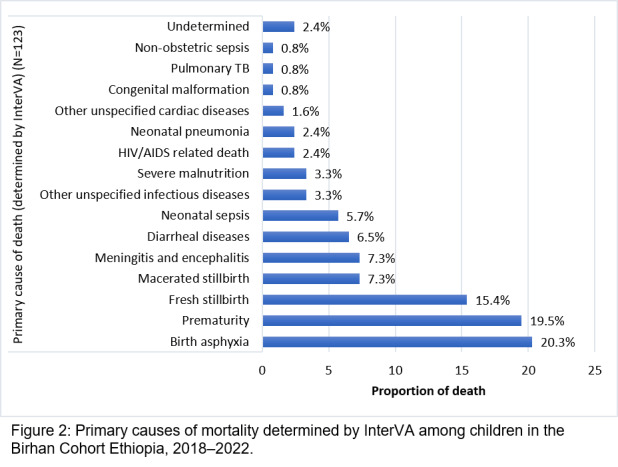
Primary causes of mortality determined by InterVA among children in the Birhan Cohort Ethiopia, 2018–22.

Of 3969 children, 135 died (mortality rate = 3.4%). Of these deaths, 129 (95.6%) occurred among infants <12 months and the rest among children 12–24 months. Of the deceased children, 97 (71.9%) had severe illness preceding death, of which 93 (95.9%) were infants and 4 (4.1%) were children 12–24 months. In contrast, 38 (28.1%) of all deaths were preceded by a mild/moderate illness. As determined by InterVA, 55.3% (n/N = 68/123) of the deaths were attributed to birth asphyxia, prematurity, and macerated stillbirth (**Figure 2**).

### The composite outcome

Among the 1397 children with illness or death, the composite outcome combining illness severity and death variables yielded 108 (7.7%) children, of which 22 survived severe illness and 86 died. The remaining 1289 (92.3%) had mild or moderate illness. Of the 92 infants who were severely ill or deceased, 76 (82.6%) were newborns and 24 (17.4%) were children 12–24 months old.

### Care-seeking

Data on the sequence of care-seeking by provider type for an illness episode were available in the VA data set, but not in the Core Child data set (*i.e.* the regular quarterly update) and Maternal and Child Health data set that captures the routine and morbidity follow-up information. As a result, the denominators for the computation of the indicators of key actions along the care pathway were not consistent. For instance, data about healthcare-seeking from a formal source were available for 1187 (84.9%) of the 1397 children who had at least one illness episode. Likewise, of those who sought care from a formal source (n = 559), only 138 (24.7%) had referral advice data, of which only 61 (44.2%) had referral acceptance data.

**Figure 3:**
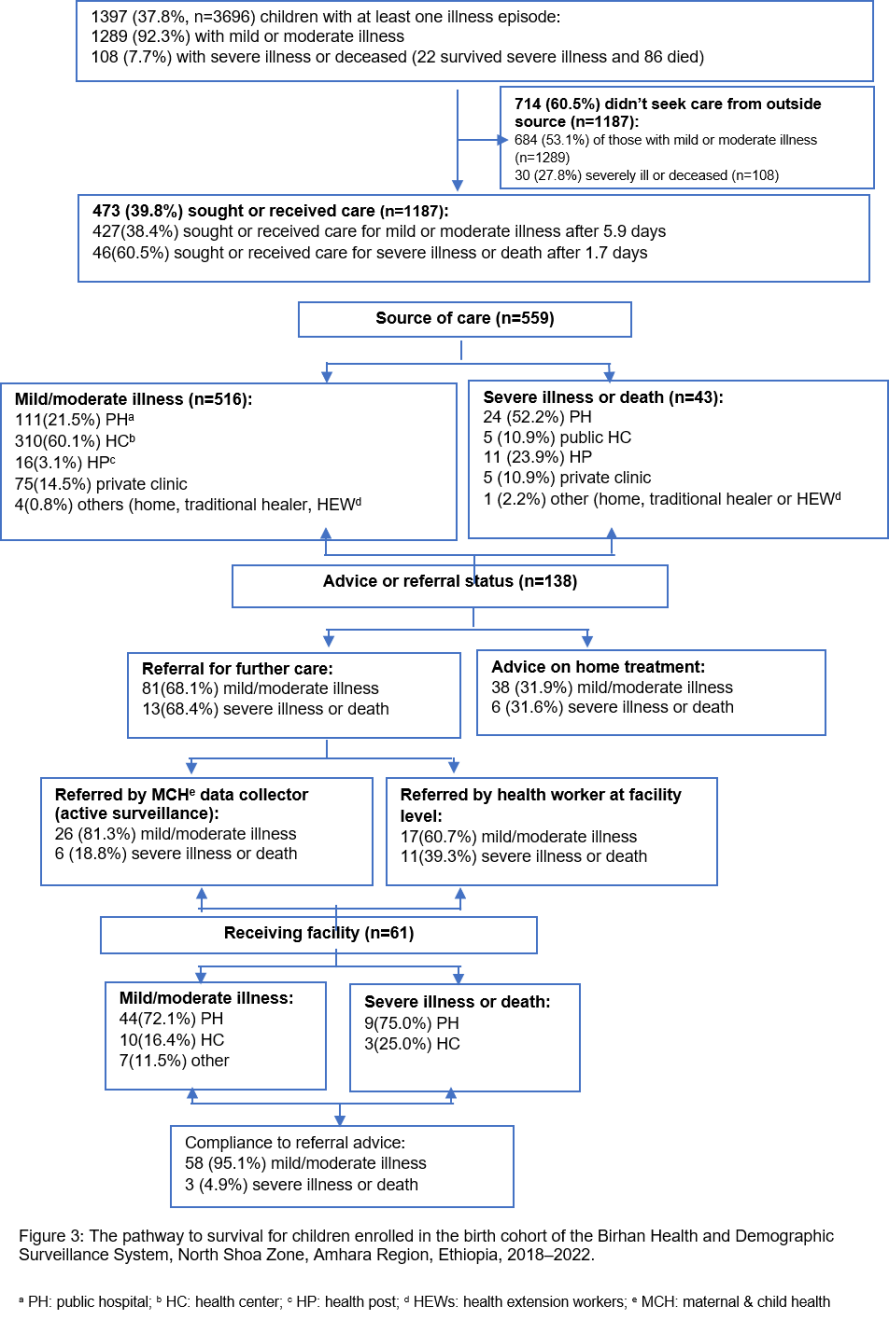
The pathway to survival for children enrolled in the birth cohort 2018–22.

Of the 1397 children with illness or death, 60.5% (n/N = 714/1187) did not get treatment from a formal source, regardless of the severity of illness (**Figure 3**). Of these, 53.1% (n/N = 684/1289) were those who had mild or moderate illness, while 27.8% (n/N = 30/108) were severely ill or deceased. None of the sick children were treated with herbal medicine. The mean delay in seeking outside care was 5.9 (standard deviation (SD) = 10.6) days after the onset of the illness for children with mild or moderate illness, and 1.7 (SD = 0.58) for severely ill or deceased children. Of 559 children, 56.1% sought care from public HCs and 24% from public hospitals. The source of outside care for 52.2% (n/N = 24/43) of the severely ill or deceased children was a public hospital, followed by an HP (n = 11; 23.9%). In contrast, 60.1% (n/N = 310/516) of caretakers of children with mild or moderate illness consulted a public HC, followed by a public hospital (n = 111; 21.5%) (**Figure 3**).

### Referral status

Of 138 children, 68.1% were referred to the next level for further care, while caretakers of 31.9% of the children were advised to provide home treatment. The primary reason that necessitated a referral was a lack of treatment modalities at the referring facility (n/N = 24/56; 42.9%), followed by the need for evaluation by a better-qualified professional or a specialist (n = 19; 33.9%). Public hospitals received 68.0% (n/N = 149/219) of the referrals, and HCs received 17.6% (n/N = 13/74). Overall, 91% (n/N = 61/67) of caretakers of referred children complied with the referral advice. However, 68.4% (n/N = 13/19) of the severely ill or deceased were referred despite the severity of their illnesses, and 4.9% (n/N = 3/61) of caretakers accepted the referral (**Figure 3**).

### Unadjusted effects of factors associated with severe childhood illness or death

Being female compared to male (OR = 0.02; 95% CI = 0.01–0.04) and being a newborn baby compared to an infant (28 days–12 months) or older child (12–24 months) (OR = 0.004; 95% CI = 0.002–0.01) were the child-related factors associated with lower odds of severe illness or death. In contrast, LBW (OR = 3.89; 95% CI = 2.27–6.69) and premature birth (OR = 2.42; 95% CI = 1.51–3.88) were associated with higher odds of severe illness or death. A mother’s attendance at ≥1 antenatal care visit was associated with a reduced risk of severe illness or death (OR = 0.60; 95% CI = 0.04–0.91), whereas experiencing complications during labour or delivery of the index baby increased the odds of this outcome (OR = 3.47; 95% CI = 1.52–7.94). Among other family- or household-related factors, the unadjusted analysis showed a protective effect on severe illness or death for children from Kewet compared to Angolela Tera *woreda* (OR = 0.34; 95% CI = 0.23–0.51), those from middle income (OR = 0.42; 95% CI = 0.27–0.68) or rich (OR = 0.37; 95% CI = 0.23–0.61) compared to poor households, and those whose caregivers sought care from a HC (OR = 0.08; 95% CI = 0.03–0.20) or a private clinic (OR = 0.31; 95% CI = 0.11–0.84) compared to a government hospital. However, seeking care from a HP was associated with higher odds of severe illness or death (OR = 3.18; 95% CI = 1.31–7.71) compared to a government hospital. Similarly, walking ≥30-minute compared to <30-minute to reach the nearest HC or hospital (OR = 2.38; 95% CI = 1.42–4.01) and being referred by a health worker compared to not being referred (OR = 40.50; 95% CI = 15.17–104.60) were associated with higher odds of severe illness or death (Table S1 in the **Online Supplementary Document**).

### Adjusted effects of factors associated with severe childhood illness or death

Being an infant (28–60 days old) (adjusted OR (aOR) = 0.05; 95% CI = 0.008–0.27) and being a child (12–24 months old) (aOR = 0.03; 95% CI = 0.005–0.17) were associated with lower odds of experiencing severe illness or death compared to a newborn baby (0–28 days old). After adjustment, the preventive effects of having a mother who completed ≥1 antenatal care visit or sought care from a HC, as well as the increased risk associated with obstetric complications during labour and delivery, were no longer observed. Seeking care from an HP was associated with an increase in the odds of the outcome. Sick children who sought care from an HP had higher odds of severe illness or death than those who consulted a government hospital (aOR = 19.64; 95% CI = 2.71–142.40). Belonging to a rich family resulted in a reduction in the odds of the outcome compared to belonging to a poor household (aOR = 0.15; 95% CI = 0.02–0.94) (**Table 3**).

**Table 3:** Adjusted effects of factors associated with severe illness or death in the Birhan Cohort in Ethiopia, 2018–22

Exposure factors	aOR (95% CI)	*P*-value
Female sex	1.42 (0.40–5.00)	0.584
LBW *(ref: ≥2500gm)*	0.65 (0.13–3.27)	0.600
Prematurity *(ref: g*estational age *≥37 weeks)*	0.40 (0.08–2.02)	0.266
Age group *(ref: newborn)*		
Infant (28 days–12 months)	0.05 (0.008–0.27)	0.001
Older child (12–24 months)	0.03 (0.005–0.17)	0.000
Mother had ANC visit(s) during pregnancy of index baby	1.18 (0.34–4.16)	0.787
Mother had complication during labor/delivery of index baby	0.37 (0.04–3.14)	0.363
Sought care from any source	3.42 (0.53–22.14)	0.197
Type of care provider* (ref: government hospital)*		
Government HC	0.24 (0.05–1.12)	0.069
Health post	19.64 (2.71–142.4)	0.003
Private clinic	3.84 (0.52–28.32)	0.187
Kewet Woreda *(ref: Angolela Tera)*	0.27 (0.05–1.54)	0.14
Wealth group *(reference: poor)*		
Medium	0.64 (0.09–4.36)	0.646
Rich	0.15 (0.02–0.94)	0.043
≥30 minutes walking time to reach the nearest HC or hospital *(ref: <30 minutes)*	0.56 (0.14–2.32)	0.426

aOR – adjusted odds ratio, CI – confidence interval, HC – health centre, ref – reference

## DISCUSSION

We demonstrated that one in four children aged >2 years experienced at least one episode of illness in the Birhan Cohort. One in every 13 ill children encountered severe illness or death, with more than two-thirds of the burden occurring among newborns. More than two-thirds of the deceased children had severe illness preceding death. Approximately two-thirds of children had mild or moderate illness, and more than a third were severely ill or died. Care was not sought for most children. Of all children who sought formal care, more than half did so from a government HC, and the remainder from a HP. It took approximately two days to seek care after the onset of a severe illness or an illness that led to death, and approximately five days for a mild or moderate illness. The majority were referred for further care, and the absence of the required treatment was the most typical referral indication. Most caretakers usually complied with the referral advice. However, about a third of the severely ill or deceased children were not referred for further care, and among those who were referred, a few caretakers accepted the advice.

These findings highlight gaps in the recognition of markers of severe illness resulting in delayed healthcare-seeking. Bypassing HPs and seeking care from HCs and hospitals may suggest a lack of knowledge of service availability or perceived low quality of care provided by health extension workers and a lack of confidence in their competence. This may lead to inefficiency from both the provider and caretaker perspectives. Supply-side barriers to care provision, including gaps in the readiness of health facilities and adherence of providers to referral guidelines, are also evident from the pathway analysis. Low care-seeking behaviour coupled with delays when care was sought, and low referral rate of severely ill children, implies the likelihood of illness progression leading to death. The findings suggest that modifiable factors might exist at individual, family, community, and health facility levels.

Our findings are consistent with previous studies from Ethiopia that revealed gaps in illness recognition and care-seeking behaviours [2,7,20]. As noted by Koffi et al [15], this might be due to low health literacy in the community. A low care-seeking practice could also suggest that caretakers might not realise the importance of signs and symptoms that signal severe illness necessitating immediate care [20,21]. Amouzou et al. [22] have demonstrated inadequate demand creation efforts for integrated community case management interventions in Ethiopia.

We also found that HPs were less frequently consulted by caretakers compared to HCs and hospitals. Several studies conducted in Ethiopia have reported similar findings, with low-quality care (both technical and interpersonal) provided at this level as a major reason [9,10,23–26]. For instance, Shimelis et al. [27] reported that half of the caretakers bypassed the nearest primary healthcare facility.

The delays in seeking formal healthcare that we observed are supported by a similar study conducted in Tanzania [28], particularly for mild or moderate illness. However, the mean delay of about two days (1.7) for severe illness is slightly longer than the 1.3 days reported in Nigeria [17]. This might be because they analysed only deceased children, while we considered both deceased and survivors of severe illness. The delays in care-seeking may suggest that caretakers might have sought informal care first, which caused the delay [28]. However, each of the forms we used would have a time/date stamp to determine the sequence of care. The delay could also be related to a lack of knowledge about the danger signs and the appropriate place to seek care [16], associated costs, and lack of transportation. Any barrier to timely care-seeking is likely to enhance illness progression, leading to death [28].

The low referral rate of severely ill or deceased children and the low acceptance rate of those who were referred have also been noted in previous studies conducted in Ethiopia [9,29,30] and other sub-Saharan African countries [7,21]. Evidence shows that compliance of healthcare providers with referral guidelines is suboptimal [30], due to knowledge and skill gaps [9,30]. Caretakers’ acceptance of referral advice is mainly constrained by lack of transport, unaffordable costs, and long distance to the referral facility [7,9,30]. Lack of confidence in the quality of care at the receiving facility has also been reported as a barrier [30]. However, this needs to be explored further.

We also found that the age of the child, type of care provider, and wealth status were the exposure factors that showed significant effects on the risk of severe childhood illness or death. Being a newborn, seeking care from an HP, and belonging to a poor household were significantly associated with an increased risk of severe childhood illness or death. The positive associations between younger age (newborn) and poor wealth status, and the risk of severe childhood illness or death are also evident in the literature [3,7,31]. Although weak, given the wider CI, there was evidence of increased odds of severe illness or death when care was sought from an HP. This could be due to low-quality care provided at this level, as previously noted [9,10,23–26]. All these findings imply the existence of modifiable factors at the family and healthcare facility level.

Our study has strengths and limitations. First, we applied the PSCF [8] as a tool for developing and monitoring integrated child survival programs. The analysis has generated up-to-date and comprehensive evidence in terms of modifiable behavioural and health systems factors for policymakers and practitioners to improve child survival programs, such as integrated management of newborn and childhood illness and integrated community case management. Potential areas for further investigation have also been identified. However, the framework assumes that care provided both at the community and health facility level is of acceptable quality and thus is limited in its ability to assess care quality [8]. Second, we used multiple data sets on children and their caretakers. This has contributed to the comprehensive understanding of the pathway to child survival programs in the study area. However, this resulted in missing data and their exclusion from further analysis, which might have biased the results. To minimise this, we employed a complete case analysis approach for multivariate analysis. Third, many of the variables we used, including the dependent variable, were composites, and this facilitated summarisation of complexities related to the use of longitudinal data. However, this might have led to the loss of important information. As a result, illness recognition, care-seeking, and provision practices might have been underestimated due to the aggregation of observations at multiple time points into one composite indicator for each action taken along the pathway. This assumed that care-seeking and provision experiences remained the same across all time points a child might have had during the follow-up period. However, this may not have always been the case. A child may have experienced multiple illness episodes and could have had several consultations for the same episode, either with the same provider or different types of providers. Furthermore, a child with mild illness at one time point could have experienced severe illness at later time point and might have subsequently died. Caretakers’ experiences from all these encounters are likely to vary. Disaggregation by several time points was not feasible due to the limited number of observations in each time point, which hindered the ability to draw meaningful conclusions. The finding that over two-thirds of deceased children experienced severe illness before death supports the assumption that the mechanisms leading to severe illness and death are similar [15], and therefore, the composite outcome we used is valid.

## CONCLUSIONS

We found that illness recognition and care-seeking for children were low, and when care was sought, it was delayed. Care-seeking from HPs was lower than from HCs and hospitals, and referral rates were low despite high overall compliance. About a third of severely ill or deceased children were not referred, often due to the unavailability of required treatment. The risk of severe illness or death was higher among newborns than older children, those who sought care from HPs rather than hospitals, and those from poor rather than rich families.

Strategies to improve child survival should target modifiable factors at individual, family, community, and health system levels. Caretakers’ recognition of illnesses should be enhanced through tailored education, community engagement programmes, and demand creation using the existing platforms. Health facilities, particularly HPs, need strengthened capacity to manage sick children. Health workers should counsel caretakers on danger signs and adhere to referral guidelines. Newborn health monitoring should be strengthened through active surveillance for early detection of fatal conditions and timely management.

Further research is needed to understand delayed or non-existent care-seeking, the quality of care provided, and the reasons for bypassing HPs. Studies should also explore experiences with preventive care, such as exclusive breastfeeding and vaccination. Longitudinal studies and birth cohorts should be designed to capture each step in the care pathway, including referral linkages and the role of community health volunteers in linking sick children with formal care providers.

## Additional material


Online Supplementary Document
