# Supplementary material for: The pathway to child survival in the Birhan Cohort, Ethiopia, 2018–22
Source: J Glob Health. 2025 Oct 3;15:04270. doi: 10.7189/jogh.15.04270 (PMC12491907; doi:10.7189/jogh.15.04270)
Supplement: Online Supplementary Document [file jogh-15-04270-s001.pdf]

Supplement to: Bayou N, Nigatu T, Tesfaye B, Hunegnaw B, Pons-Duran C, Alemu K, Tadesse L, Bekele D, Tollera G, Chan GJ. The pathway to child survival in the Birhan Cohort, Ethiopia, 2018–2022. J Glob Health. 2025;15:04270.

Table S1: Unadjusted effects of factors associated with severe childhood illness or death in the Birhan Cohort in Ethiopia, 2018–2022.

Exposure factors	COR	P-value	95% CI
**Child related**			
Female sex	0.65	**0.036**	**0.43, 0.97**
Age group *(Reference: Newborn)*			
Infant (28 days – 12 months)	0.02	**0.000**	**0.01, 0.04**
Older child (12-24 months)	0.004	**0.000**	**0.002, 0.01**
LBW *(Reference: >= 2500gm)*	3.89	**0.000**	**2.27, 6.69**
Community birth *(Reference: Facility birth)*	0.89	0.535	0.52, 1.41
Prematurity *(Reference: Gestational age >= 37 weeks)*	2.42	**0.000**	**1.51, 3.88**
**Mother, caretaker or family and household related**			
Had any ANC visit during pregnancy of index baby	0.60	**0.015**	**0.40, 0.91**
Had history of past medical problem	0.63	0.096	0.36, 1.09
Had any problem in prior pregnancy/birth	3.83	0.084	0.83, 17.59
Had complication during labor or delivery of index baby	3.47	**0.003**	**1.52, 7.94**
Informal or no education *(Reference: Formal education)*	1.45	0.070	0.97, 2.15
Age *(Reference: Below 20 years)*			
20-29 years	0.51	0.051	0.26, 1.003
30 years and above	0.72	0.343	0.37, 1.41
Kewet Woreda *(Reference: Angolela Tera)*	0.34	**0.000**	**0.23, 0.51**
Rural residence *(Reference: Urban)*	1.45	0.144	0.88, 2.40
Larger family size, i.e., >=4 *(Reference: 1-3)*	1.15	0.542	0.73, 1.81
Wealth group *(Reference: Poor)*			
Meddle income	0.42	**0.000**	**0.27, 0.68**
Rich	0.37	**0.000**	**0.23, 0.61**
Sought care from any source	2.46	**0.000**	**1.53, 3.95**
Type of care provider *(Reference: Hospital)*			
Health center	0.08	**0.000**	**0.03, 0.20**
Health post	3.18	**0.010**	**1.31, 7.71**
Private clinic	0.31	**0.022**	**0.11, 0.84**
Other (home, traditional healer, HEW)	1.16	0.899	0.12, 10.81
**Health system related**			
>=30 minutes walking time to reach the nearest HC or hospital (Reference: <30 minutes)	2.38	**0.001**	**1.42, 4.01**
Referred by health worker at health facility	40.50	**0.000**	**15.7, 104.6**
Advised on referral	1.14	0.762	0.49, 2.69
